# Sustained high Life’s Essential 8 is associated with lower risk of cerebral small vessel disease: a community-based study

**DOI:** 10.3389/fneur.2025.1563288

**Published:** 2025-07-09

**Authors:** Huijing Shi, Liufu Cui, Ying Hui, Rong Shu, Haicheng Song, Jierui Wang, Ping Yu, Shuohua Chen, Jing Li, Xiaoliang Liang, Chunyu Ruan, Jing Chen, Zhenchang Wang, Shouling Wu, Xiaoshuai Li

**Affiliations:** ^1^Department of Rheumatology and Immunology, Kailuan General Hospital, Tangshan, China; ^2^Department of Radiology, Kailuan General Hospital, Tangshan, China; ^3^Department of Cardiology, Kailuan General Hospital, Tangshan, China; ^4^Department of Radiology, Beijing Tsinghua Changgung Hospital, Beijing, China; ^5^Department of Psychiatry, Kailuan Mental Health Centre, Tangshan, China; ^6^Department of Radiology, Beijing Friendship Hospital, Capital Medical University, Beijing, China

**Keywords:** Life’s Essential 8, cardiovascular health, cerebral small vessel disease, trajectories, risk

## Abstract

**Objective:**

We aimed to investigate the influence of Life’s Essential 8 (LE8) score trajectories on cerebral small vessel disease (CSVD).

**Methods:**

A total of 1,162 subjects from the Kailuan prospective cohort study were enrolled. The LE8 score trajectories were developed from 2006 to 2021 using trajectory modeling of the SAS procedure Proc Traj. Participants were classified into three trajectory models: low stable group, medium stable group, and high stable group. Magnetic resonance imaging (MRI) was performed at the last follow-up. Ordered logistic regression and binary logistic regression models were employed to analyze the association between LE8 trajectory groups and the total CSVD score. Furthermore, we examined the relationship between LE8 trajectory groups and Various CSVD Lesions: lacunar infarcts, cerebral microbleeds (CMB), white matter hyperintensities (WMH), and enlarged perivascular spaces (EPVS).

**Results:**

(1) There is a negative association between sustained high LE8 scores and the risk of CSVD. The odds ratios (OR) for an increased risk of elevated CSVD scores in the medium and high stable groups compared to the low stable group were 0.65 (95% CI: 0.47–0.88) and 0.44 (95% CI: 0.30–0.63), both of which were statistically significant. (2) LE8 had the most significant impact on lacunar infarcts, EPVS and WMH. The ORs for an increased risk of lacunar infarcts in the medium and high stable groups compared to the low stable group were 0.56 (95% CI: 0.37–0.86) and 0.25 (95% CI: 0.14–0.47), respectively. The ORs for an increased risk of BG-EPVS were 0.49 (95% CI: 0.30–0.81) and 0.31 (95% CI: 0.18–0.54), respectively. The ORs for an increased risk of WMH were 0.49 (95% CI: 0.64–1.38) and 0.58 (95% CI: 0.36–0.95), respectively. However, LE8 showed no statistically significant impact on CMB.

**Conclusion:**

In summary, our study revealed that sustained high Life’s Essential 8 scores have a protective effect against cerebral small vessel disease.

## 1 Introduction

Cerebral small vessel disease (CSVD) is a syndrome characterized by clinical, imaging, and pathological manifestations (1–3). Magnetic resonance imaging features of CSVD include white matter hyperintensities (WMH), lacunar infarcts, cerebral microbleeds (CMB), and enlarged perivascular spaces (EPVS). Quite common in the general population, CSVD affects approximately 5% of adults over 50 years old and nearly 100% of those over 90 years old (4). Epidemiological data on CSVD indicate that the median prevalence of moderate to severe WMH, lacunar infarcts, CMB, and EPVS in global community populations is 20.5, 0.8, 10.7, and 25.0%, respectively (5). The clinical manifestations of CSVD are diverse and complex, including symptoms such as cognitive impairment, motor disorders, emotional disturbances, and reduced social functioning, making CSVD a major risk factor for various acute and chronic neurological diseases (6). It ultimately leads to decreased activities of daily living for patients (7).

Among the numerous risk factors for CSVD, aging and hypertension are the most clearly defined, with diabetes, smoking, excessive alcohol consumption, hypercholesterolemia, and stroke also identified as risk factors (8). However, previous studies have primarily focused on the impact of single factors on CSVD without considering the combined effects and interactions of multiple factors. In 2022, the American Heart Association (AHA) introduced the definition and standards for Life’s Essential 8 (LE8), recommending it as a measure of cardiovascular health (CVH) (9). Several studies have shown that as LE8 scores increase, the risks of cardiovascular diseases and cerebrovascular events decrease (10, 11). However, no research has yet examined the relationship between LE8 scores and different sustained states with CSVD. Therefore, we utilized data from the Kailuan Study to explore the relationship between the scoring trajectories defined by the new LE8 indicators and CSVD.

## 2 Materials and methods

### 2.1 Subjects

From July 2006 to October 2007, a health examination involving a total of 101,510 participants was conducted for employees of the Kailuan Group at Kailuan General Hospital and 11 affiliated hospitals. Follow-up examinations were conducted for the same cohort during the periods of 2008–2009, 2010–2011, 2012–2013, 2014–2015, 2016–2017, and 2018–2019, resulting in a total of seven follow-ups to monitor relevant indicators. As of 2020, all seven follow-ups have been completed. In the 2020 follow-up (the eighth), participants were voluntarily recruited for observation and underwent free cranial MRI scans for the diagnosis of cerebral small vessel disease.

### 2.2 Inclusion and exclusion criteria

**Figure 1:**
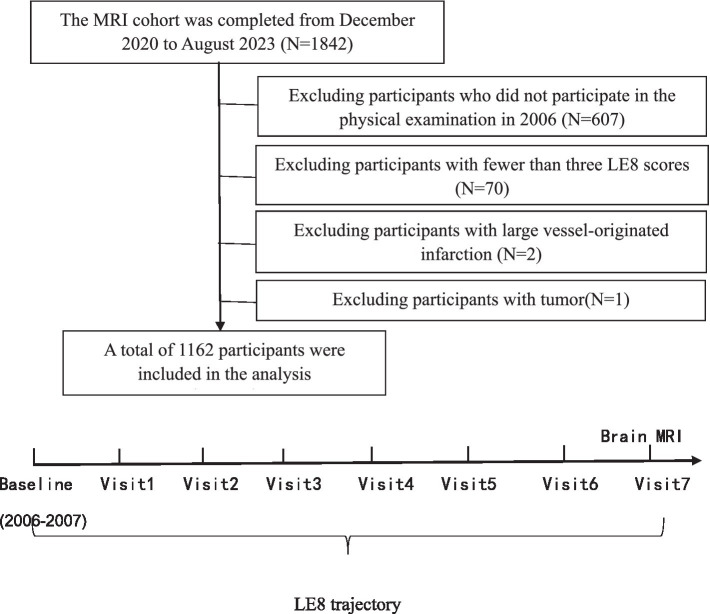
Flow chart of the current study.

Inclusion criteria: (1) Age ≥18 years old. (2) Participants who agreed to take part in the Kailuan Study and undergo cranial MRI scans and signed the informed consent form. Exclusion criteria: (1) Contraindications for MRI (including individuals who have undergone heart, brain, or kidney revascularization procedures, major arterial stenting, have cardiac pacemakers or valve replacements, possess metal implants, and pregnant or lactating women). (2) Individuals who did not participate in the 2006 health examination. (3) Participants who missed three or more of the eight follow-ups. (4) Individuals with large vessel-related cerebral infarction. (5) Individuals with tumors (see Figure 1).

### 2.3 Data collection and relevant definitions

#### 2.3.1 General information collection

Information on sociodemographic and lifestyle factors was collected via questionnaires, including age, sex, drinking status, education level, sleep duration, physical activity. Medical conditions (hypertension, diabetes, dyslipidemia) and medication use were obtained via self-reported questionnaires, medical records, and physical examinations. Measurements of height, weight, and blood pressure parameters were done by trained physicians, following standardized protocols. Current drinkers were defined as an average daily intake of 100 mL of liquor (with an alcohol content greater than 50%) over the past year. Education level was defined as having completed high school or higher.

The fasting blood samples were tested using a Hitachi 747 auto-analyzer (Hitachi, Tokyo, Japan) at the central laboratory of the Kailuan Hospital, including fasting blood glucose (FBG), total cholesterol (TC), and high-density lipoprotein cholesterol (HDL-C) non-HDL-C = TC-HDL-C.

The contents of the epidemiological survey, and anthropometric and biochemical evaluations are described in greater detail elsewhere (12).

#### 2.3.2 LE8 score

The LE8 score was assessed using the algorithm specified by the American Heart Association (AHA), which includes four health behaviors (diet, physical activity, smoking, and sleep) and four health factors (body mass index, non-high-density lipoprotein cholesterol, fasting blood glucose, and blood pressure). Each factor is scored from 0 to 100. The overall cardiovascular health (CVH) score for each participant is calculated by summing the scores of all eight indicators and dividing by 8. Due to the lack of detailed dietary data, and considering the effects of salt intake, tea consumption, and high-fat diets on cardiovascular disease risk in the Chinese population, we used the unweighted average of the scores for salt intake (0–100), frequency of tea consumption (0–100), and high-fat food intake (0–100) as a substitute for the AHA’s DASH-style dietary score. This alternative method has been widely recognized in the field and utilized in relevant studies (10, 11, 13) (detailed in Supplementary Table S1).

#### 2.3.3 MRI image collection

MRI images were acquired using a 3.0 T MRI scanner (MR750w, General Electric, Waukesha, WI, United States) in the MRI room of the Kailuan General Hospital. The sequences used in the MRI examination included the following: T2-weighted imaging (T2WI), three-dimensional (3D) brain volume (BRAVO) high-resolution T1-weighted imaging (T1WI), fluid-attenuated inversion recovery (FLAIR), diffuse-weighted imaging (DWI, *b* = 0 and *b* = 1,000) and susceptibility-weighted angiography (SWAN). Brain imaging data were acquired in the Digital Imaging and Communications in Medicine (DICOM) format. The protocols for MRI are detailed in Supplementary Table S2. All images were independently evaluated by two experienced neuroradiologists. The two neuroradiologists (Yinghui and Jingli) independently evaluated the images of 100 participants to ensure internal consistency of image interpretation in the present study, the *κ* coefficients were 0.72–0.88, respectively. Discrepancies in image interpretation were resolved through consultation and discussion with a third higher-level physician.

#### 2.3.4 Total burden score of cerebral small vessel disease

According to the *Neuroimaging Vascular Lesion Reporting Standards* for CSVD research (14), four types of CSVD lesions were identified: lacunar infarcts, CMB, EPVS, and WMH. The severity of CSVD was assessed using two methods: the CSVD score (15) and the modified CSVD score (16). The specific scoring criteria are as follows: CSVD total score: One point is assigned for any lacunar infarct, one point for the presence of CMB, one point for severe EPVS (>10) in the basal ganglia, and one point if the Fazekas score for periventricular WMH is 3 and/or the Fazekas score for deep WMH is ≥2, resulting in a total CSVD score ranging from 0 to 4. Modified CSVD total score: One point for any lacunar infarct, one point for severe EPVS (>20) in the basal ganglia, one point for 1–4 CMB, two points for ≥5 CMB, one point if the WMH burden (periventricular + deep WMH Fazekas score) is rated 3–4, and two points if rated 5–6, resulting in a modified CSVD score ranging from 0 to 6.

### 2.4 Statistical methods

Statistical analysis was performed using SAS 9.4. Continuous, normally distributed variables were presented as the mean ± standard deviation (SD), and groups were compared using one-way ANOVA. Category variables were presented by number and percentage (%), with comparisons between groups by chi-square test.

Trajectory modeling was performed using the SAS Proc Traj program, incorporating the eight LE8 scores into the trajectory modeling. LE8 was modeled as a continuous variable using CNORM distribution. The analysis proceeded as follows: first, the optimal number of trajectory groups was determined by evaluating models with 2 to 5 groups. Subsequently, polynomial orders (linear, quadratic, or cubic terms) were specified for each trajectory group. Model selection adhered to the following criteria: (1) minimization of the Bayesian information criterion (BIC); (2) average posterior probability (AvePP) >70% for all groups; and (3) adequate group sample sizes (>5.0% of the cohort). Among candidate models, the 3-group and 4-group solutions demonstrated comparable BIC values. Given that there were only 128 participants in our high stable group, it was not considered conducive to model analyses of those in groups, therefore, the 3-group model with quadratic polynomial orders (2-2-2) was selected as optimal, satisfying all statistical thresholds (BIC = −23,242; AvePP: 0.90–0.93; group proportions: 18.2, 44.5, 37.3%).

Ordered logistic regression and binary logistic regression models were employed to analyze the association between LE8 trajectory groups and the total CSVD score, as well as the incidence of events defined as ≥1 and ≥2. The same models were used to evaluate the relationship between LE8 trajectory groups and lacunar infarcts, CMB, WMH, and EPVS. Considering the impact of gender, age, education level, and alcohol consumption on cardiovascular health and CSVD, two models were constructed. Model one was a univariate analysis, while model two adjusted for gender, baseline age, education level, and alcohol consumption history. A *p*-value of <0.05 (two-sided) was considered statistically significant.

To further demonstrate that LE8 trajectory scoring provides a better assessment of the association with cerebral small vessel disease compared to a single LE8 score, we categorized participants based on their LE8 scores from 2006 and 2020 into three groups: low (0–49), medium (50–79), and high (80–100). The impact of single LE8 score groupings on CSVD and its various lesions was analyzed, with the adjustment models consistent with those described above.

### 2.5 Exploratory analysis

To further analyze the correlation between LE8 and CSVD, we conducted a stratified analysis by gender. Finally, we separately analyzed the impacts of health behaviors trajectories and health factors trajectories on CSVD.

## 3 Results

From December 2020 to August 5, 2023, a total of 1,842 subjects underwent imaging and related examinations. After excluding 607 individuals who did not participate in the 2006 health examination, 70 individuals with fewer than three LE8 scores, 2 with large vessel-originated infarction, and 1 with a tumor, 1,162 subjects were included in the final statistical analysis.

### 3.1 General information

**Figure 2:**
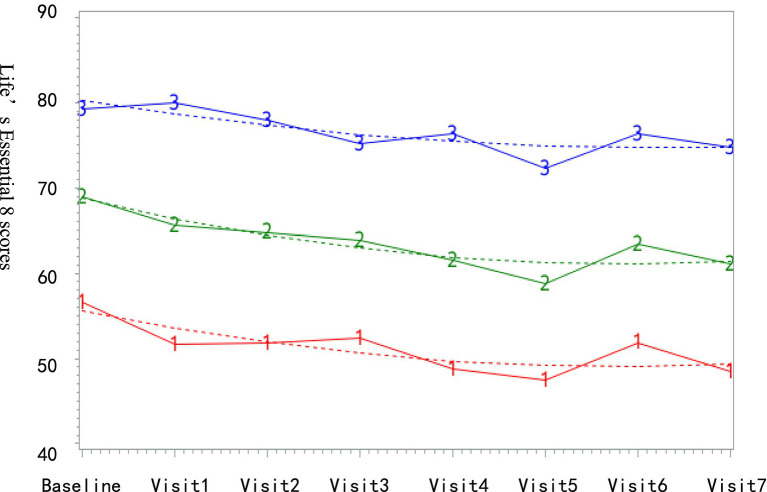
Life’s Essential 8 trajectories.

The final 1,162 subjects had an average follow-up of 5.4 ± 1.6 times, with an average age of 42.6 ± 9.9 years. There were 575 males (49.5%). Based on LE8 trajectory grouping, as shown in Figure 2, the low-stable group comprised 211 subjects (18.2%) with LE8 scores ranging from 47.06 to 56.45; the medium-stable group included 517 subjects (44.5%) with LE8 scores between 58.69 and 68.93; and the high-stable group consisted of 434 subjects (37.3%) with LE8 scores fluctuating from 72.26 to 80.18.

General characteristics of the study population are displayed in Table 1. The CSVD score distribution in the entire population ranged from 0 to 4 points, with proportions of 25.7% (299), 36.5% (424), 20.4% (237), 10.3% (120), and 7.1% (82), respectively. The modified CSVD score distribution from 0 to 6 points was as follows: 51.6% (600), 22.8% (265), 11.6% (135), 6.6% (77), 3.7% (43), 2.3% (27), and 1.3% (15). Compared to the low-stable group, the high-stable group had younger subjects (38.1 ± 8.5 years), a lower proportion of males (20.3%), higher education levels, and lower proportions of subjects with a history of alcohol consumption and medication use.

**Table 1:** Baseline characteristics of participants.

Variable	Total (*N* = 1,162)	Low-stable group (*N* = 211)	Medium-stable group (*N* = 517)	High-stable group (*N* = 434)	*p*
Age (years)	42.6 ± 9.9	45.6 ± 8.9	45.2 ± 10.0	38.1 ± 8.5	<0.001
Male [*n* (%)]	575 (49.5)	185 (87.7)	302 (58.4)	88 (20.3)	<0.001
BMI (kg/m^2^)	24.5 ± 3.4	27.1 ± 3.2	25.2 ± 3.0	22.3 ± 2.6	<0.001
FBG (mmol/L)	5.2 ± 1.3	5.6 ± 1.7	5.2 ± 1.4	4.9 ± 0.6	<0.001
TC (mmol/L)	4.9 ± 0.9	5.2 ± 1.1	5.0 ± 0.9	4.6 ± 0.8	<0.001
HDL-C (mmol/L)	1.5 ± 0.4	1.5 ± 0.4	1.5 ± 0.4	1.5 ± 0.3	0.053
Non-HDL-C (mmol/L)	3.4 ± 0.9	3.8 ± 1.1	3.4 ± 0.9	3.1 ± 0.8	<0.001
SBP (mmHg)	117.9 ± 16.4	129.2 ± 17.0	120.9 ± 15.1	108.8 ± 12.4	<0.001
DBP (mmHg)	77.7 ± 10.3	84.2 ± 10.1	79.6 ± 9.8	72.3 ± 8.1	<0.001
Sleep duration (H)	7.4 ± 2.0	7.0 ± 1.1	7.5 ± 2.8	7.6 ± 0.8	0.001
Sodium intake [*n* (%)]					0.001
<6 g/day	99 (8.5)	18 (8.5)	44 (8.5)	37 (8.5)	
6–10 g/day	949 (81.7)	157 (74.4)	422 (81.6)	370 (85.3)	
>10 g/day	114 (9.8)	36 (17.1)	51 (9.9)	27 (6.2)	
High-fat food intake [*n* (%)]					<0.001
<1 time/week	88 (7.6)	8 (3.8)	42 (8.1)	38 (8.8)	
1–3 time/week	959 (82.5)	163 (77.3)	430 (83.2)	366 (84.3)	
>3 time/week	115 (9.9)	40 (19.0)	45 (8.7)	30 (6.9)	
Tea intake [*n* (%)]					0.002
Never	725 (62.4)	126 (59.7)	339 (65.6)	260 (59.9)	
<1 time/month	106 (9.1)	16 (7.6)	34 (6.6)	56 (12.9)	
1–3 times/month	123 (10.6)	23 (10.9)	56 (10.8)	44 (10.1)	
1–3 times/week	92 (7.9)	15 (7.1)	34 (6.6)	43 (9.9)	
≥4 times/week	116 (10.0)	31 (14.7)	54 (10.4)	31 (7.1)	
Physical activity [*n* (%)]					<0.001
Never	87 (7.5)	27 (12.8)	39 (7.5)	21 (4.8)	
1–3 times/week	964 (83.0)	164 (77.7)	417 (80.7)	383 (88.2)	
≥4 times/week	111 (9.6)	20 (9.5)	61 (11.8)	30 (6.9)	
Smoking status [*n* (%)]					<0.001
Never	877 (75.5)	76 (36.0)	386 (74.7)	415 (95.6)	
Former smokers quit ≥1 year	46 (4.0)	10 (4.7)	28 (5.4)	8 (1.8)	
Current <1 cigarette/day	32 (2.8)	11 (5.2)	19 (3.7)	2 (0.5)	
Current ≥1 cigarette/day	207 (17.8)	114 (54.0)	84 (16.2)	9 (2.1)	
CSVD score					<0.001
0	299 (25.7)	24 (11.4)	99 (19.1)	176 (40.6)	
1	424 (36.5)	71 (33.6)	170 (32.9)	183 (42.2)	
2	237 (20.4)	50 (23.7)	135 (26.1)	52 (12.0)	
3	120 (10.3)	36 (17.1)	66 (12.8)	18 (4.1)	
4	82 (7.1)	30 (14.2)	47 (9.1)	5 (1.2)	
Modified CSVD score					<0.001
0	600 (51.6)	83 (39.3)	216 (41.8)	301 (69.4)	
1	265 (22.8)	36 (17.1)	137 (26.5)	92 (21.2)	
2	135 (11.6)	39 (18.5)	71 (13.7)	25 (5.8)	
3	77 (6.6)	22 (10.4)	45 (8.7)	10 (2.3)	
4	43 (3.7)	16 (7.6)	22 (4.3)	5 (1.2)	
5	27 (2.3)	12 (5.7)	14 (2.7)	1 (0.2)	
6	15 (1.3)	3 (1.4)	12 (2.3)	0 (0)	
High school or above [*n* (%)]	601 (53.7)	89 (46.4)	229 (46.1)	283 (65.7)	<0.001
Current drinker [*n* (%)]	422 (36.7)	141 (67.5)	198 (38.9)	83 (19.2)	<0.001
Lipid-lowering drugs [*n* (%)]	7 (0.7)	1 (0.6)	5 (1.1)	1 (0.2)	0.305
Diabetes history [*n* (%)]	16 (1.4)	8 (4.2)	8 (1.6)	0 (0)	<0.001
Antihypertensive drugs [*n* (%)]	93 (8.4)	45 (23.9)	45 (9.2)	3 (0.7)	<0.001

BMI, body mass index; TC, total cholesterol; HDL-C, high-density lipoprotein cholesterol; non-HDL-C, non-high-density lipoprotein cholesterol; FBG, fasting blood glucose; SBP, systolic blood pressure; DBP, diastolic blood pressure; CSVD, cerebral small vessel disease.

### 3.2 Impact of different LE8 groups on CSVD

The LE8 trajectory score exhibits a dose-response relationship with the risk of CSVD. The results of the ordered logistic regression analysis showed that, after adjusting for the effects of gender, baseline age, alcohol consumption, and education level, the odds ratios (OR) for an increased risk of elevated CSVD scores in the medium and high stable groups compared to the low stable group were 0.65 (95% CI: 0.47–0.88) and 0.44 (95% CI: 0.30–0.63), respectively. The ORs for an increased risk of elevated modified CSVD scores were 0.77 (95% CI: 0.56–1.05) and 0.52 (95% CI: 0.35–0.76), both of which were statistically significant. Moreover, the sustained LE8 score demonstrates a significant protective effect against moderate-to-severe CSVD. For events defined as CSVD ≥1 and modified CSVD ≥2, the high stable group maintained statistical significance compared to the low stable group, while LE8 trajectory groups had no effect on modified CSVD ≥1.

To further demonstrate that trajectory analysis provides a better assessment of LE8 scores’ impact on CSVD compared to cross-sectional analysis, we conducted a cross-sectional analysis based on LE8 scores from the 2020 health examination (the eighth follow-up), categorizing participants into low (0–49), medium (50–79), and high (80–100) groups. The results showed no statistical differences among the groups after controlling for gender, cross-sectional age, alcohol history, and education level. In contrast, when the 2006 baseline LE8 scores were categorized into low, medium, and high groups, the results differed slightly from those of the LE8 trajectory groups regarding their impact on CSVD (detailed in Table 2).

**Table 2:** Impact of different LE8 groups on CSVD.

Variable	LE8 trajectory groups & CSVD					2020 cross-sectional LE8 groups & CSVD			2006 cross-sectional LE8 groups & CSVD		
	Groups	Case/Total	Model 1	Model 2	Groups	Case/Total	Model 1	Model 2	Case/Total	Model 1	Model 2
			OR (95% CI)	OR (95% CI)			OR (95% CI)	OR (95% CI)		OR (95% CI)	OR (95% CI)
CSVD score											
	Low-stable	211	1.00	1.00	Low	105	1.00	1.00	54	1.00	1.00
	Medium-stable	517	0.65 (0.49–0.87)^*^	0.65 (0.47–0.88)^*^	Medium	530	0.67 (0.46–0.98)^*^	0.92 (0.62–1.38)	841	0.39 (0.24–0.64)^*^	0.49 (0.29–0.81)^*^
	High-stable	434	0.18 (0.13–0.25)^*^	0.44 (0.3–0.63)^*^	High	93	0.34 (0.21–0.58)^*^	0.94 (0.53–1.66)	267	0.15 (0.09–0.26)^*^	0.42 (0.23–0.75)^*^
CSVD ≥1											
	Low-stable	187/211	1.00	1.00	Low	86/105	1.00	1.00	51/54	1.00	1.00
	Medium-stable	418/517	0.54 (0.34–0.87)^*^	0.57 (0.32–0.99)^*^	Medium	383/530	0.58 (0.34–0.98)^*^	0.73 (0.39–1.4)	652/841	0.2 (0.06–0.66)^*^	0.28 (0.08–0.98)^*^
	High-stable	258/434	0.19 (0.12–0.3)^*^	0.42 (0.23–0.78)^*^	High	57/93	0.35 (0.18–0.67)^*^	0.72 (0.32–1.61)	160/267	0.09 (0.03–0.29)^*^	0.27 (0.08–1)
CSVD ≥2											
	Low-stable	116/211	1.00	1.00	Low	47/105	1.00	1.00	34/54	1.00	1.00
	Medium-stable	248/517	0.76 (0.55–1.04)	0.86 (0.59–1.26)	Medium	187/530	0.67 (0.44–1.03)	0.88 (0.52–1.47)	353/841	0.43 (0.24–0.75)^*^	0.57 (0.3–1.09)
	High-stable	75/434	0.17 (0.12–0.25)^*^	0.43 (0.27–0.68)^*^	High	17/93	0.28 (0.14–0.53)^*^	0.74 (0.33–1.62)	52/267	0.14 (0.08–0.27)^*^	0.43 (0.21–0.91)^*^
Modified CSVD score											
	Low-stable	211	1.00	1.00	Low	105	1.00	1.00	54	1.00	1.00
	Medium-stable	517	0.73 (0.55–0.98)^*^	0.77 (0.56–1.05)	Medium	530	0.74 (0.5–1.09)	0.96 (0.63–1.47)	841	0.44 (0.27–0.72)^*^	0.57 (0.34–0.95)^*^
	High-stable	434	0.22 (0.16–0.3)^*^	0.52 (0.35–0.76)^*^	High	93	0.45 (0.26–0.78)^*^	1.12 (0.6–2.07)	267	0.17 (0.1–0.28)^*^	0.44 (0.24–0.8)^*^
Modified CSVD ≥1											
	Low-stable	128/211	1.00	1.00	Low	55/105	1.00	1.00	38/54	1.00	1.00
	Medium-stable	301/517	0.9 (0.65–1.25)	1.01 (0.69–1.48)	Medium	241/530	0.76 (0.5–1.15)	0.92 (0.56–1.52)	443/841	0.47 (0.26–0.85)^*^	0.57 (0.29–1.12)
	High-stable	133/434	0.29 (0.2–0.4)^*^	0.66 (0.42–1.03)	High	35/93	0.55 (0.31–0.97)^*^	1.26 (0.63–2.52)	81/267	0.18 (0.1–0.35)^*^	0.44 (0.21–0.91)^*^

Single LE8 score was categorized as low (0–49), moderate (50–79) or high (80–100) group. ^*^p < 0.05.

### 3.3 Impact of different LE8 groups on various CSVD lesions

Logistic regression analysis results for various CSVD lesions based on LE8 trajectory groups indicated that LE8 had the most significant impact on lacunar infarcts and BG-EPVS. After controlling for confounding factors, the odds ratios for an increased risk of lacunar infarcts in the medium and high stable groups compared to the low stable group were 0.56 (95% CI: 0.37–0.86) and 0.25 (95% CI: 0.14–0.47), respectively. The ORs for an increased risk of BG-EPVS were 0.49 (95% CI: 0.30–0.81) and 0.31 (95% CI: 0.18–0.54), both statistically significant. For other lesions, including WMH, modified WMH, and modified BG-EPVS, only the ORs in the high stable group were statistically significant. However, LE8 showed no statistically significant impact on CMB and modified CMB.

The logistic regression analysis of single LE8 score groupings on various CSVD lesions revealed that the 2020 cross-sectional LE8 score was negatively correlated only with the risk of BG-EPVS, with an OR of 0.40 (95% CI: 0.19–0.68), which was statistically significant. In contrast, the 2006 LE8 score showed a significant negative correlation with the risk of lacunar infarcts and BG-EPVS, with no significant associations for other lesions (see Table 3).

**Table 3:** Impact of different LE8 groups on various CSVD lesions.

Variable		LE8 trajectory groups & various CSVD lesions			2020 cross-sectional LE8 groups & various CSVD lesions		2006 cross-sectional LE8 groups & various CSVD lesions	
	Groups	Model 1	Model 2	Groups	Model 1	Model 2	Model 1	Model 2
		OR (95% CI)	OR (95% CI)		OR (95% CI)	OR (95% CI)	OR (95% CI)	OR (95% CI)
Lacunar infarcts								
	Low-stable	1.00	1.00	Low	1.00	1.00	1.00	1.00
	Medium-stable	0.55 (0.38–0.79)^*^	0.56 (0.37–0.86)^*^	Medium	0.96 (0.54–1.72)	1.31 (0.69–2.51)	0.28 (0.16–0.49)^*^	0.3 (0.16–0.58)^*^
	High-stable	0.1 (0.06–0.17)^*^	0.25 (0.14–0.47)^*^	High	0.25 (0.08–0.78)^*^	0.77 (0.22–2.65)	0.04 (0.02–0.09)^*^	0.1 (0.04–0.26)^*^
CMB								
	Low-stable	1.00	1.00	Low	1.00	1.00	1.00	1.00
	Medium-stable	0.82 (0.59–1.14)	0.89 (0.62–1.28)	Medium	0.73 (0.46–1.14)	0.91 (0.56–1.48)	0.6 (0.34–1.04)	0.73 (0.4–1.33)
	High-stable	0.4 (0.27–0.57)^*^	0.77 (0.5–1.2)	High	0.7 (0.38–1.29)	1.49 (0.74–2.97)	0.32 (0.17–0.59)^*^	0.67 (0.33–1.32)
Modified CMB								
	Low-stable	1.00	1.00	Low	1.00	1.00	1.00	1.00
	Medium-stable	0.83 (0.6–1.15)	0.94 (0.66–1.33)	Medium	0.71 (0.46–1.1)	0.89 (0.56–1.43)	0.59 (0.34–1.02)	0.75 (0.42–1.34)
	High-stable	0.39 (0.27–0.56)^*^	0.78 (0.5–1.2)	High	0.67 (0.36–1.23)	1.44 (0.73–2.85)	0.31 (0.17–0.57)^*^	0.67 (0.34–1.31)
WMH								
	Low-stable	1.00	1.00	Low	1.00	1.00	1.00	1.00
	Medium-stable	0.91 (0.65–1.27)	0.94 (0.64–1.38)	Medium	0.86 (0.54–1.38)	0.98 (0.57–1.68)	0.95 (0.52–1.73)	1.25 (0.64–2.45)
	High-stable	0.27 (0.18–0.41)^*^	0.58 (0.36–0.95)^*^	High	0.48 (0.24–0.96)^*^	0.99 (0.44–2.21)	0.47 (0.24–0.91)^*^	1.36 (0.63–2.93)
Modified WMH								
	Low-stable	1.00	1.00	Low	1.00	1.00	1.00	1.00
	Medium-stable	0.75 (0.54–1.04)	0.73 (0.51–1.07)	Medium	0.62 (0.39–0.97)^*^	0.67 (0.4–1.12)	0.71 (0.4–1.25)	0.9 (0.48–1.68)
	High-stable	0.2 (0.13–0.31)^*^	0.44 (0.27–0.71)^*^	High	0.29 (0.14–0.62)^*^	0.6 (0.26–1.4)	0.25 (0.13–0.49)^*^	0.7 (0.33–1.49)
BG-EPVS								
	Low-stable	1.00	1.00	Low	1.00	1.00	1.00	1.00
	Medium-stable	0.48 (0.31–0.75)^*^	0.49 (0.3–0.81)^*^	Medium	0.51 (0.31–0.84)^*^	0.66 (0.37–1.21)	0.19 (0.07–0.53)^*^	0.26 (0.09–0.77)^*^
	High-stable	0.15 (0.1–0.23)^*^	0.31 (0.18–0.54)^*^	High	0.19 (0.1–0.36)^*^	0.4 (0.19–0.86)^*^	0.08 (0.03–0.21)^*^	0.23 (0.07–0.71)^*^
Modified BG-EPVS								
	Low-stable	1.00	1.00	Low	1.00	1.00	1.00	1.00
	Medium-stable	0.93 (0.65–1.31)	0.95 (0.64–1.41)	Medium	0.86 (0.53–1.38)	1.03 (0.59–1.78)	0.66 (0.37–1.19)	0.77 (0.4–1.48)
	High-stable	0.23 (0.15–0.36)^*^	0.5 (0.3–0.84)^*^	High	0.16 (0.06–0.45)^*^	0.34 (0.11–1)	0.25 (0.13–0.5)^*^	0.63 (0.29–1.37)

Lacunar infarcts was classified into grade 0–1; CMB, cerebral microbleeds, classified into grade 0–1; modified CMB was classified into grade 0–2; WMH, white matter hyperintensities, classified into grade 0–1; modified WMH was classified into grade 0–2; BG-EPVS, enlarged perivascular space in basal ganglia, classified into grade 0–1; modified BG-EPVS was classified into grade 0–1. Single LE8 score was categorised as low (0–49), moderate (50–79) or high (80–100) group. ^*^p < 0.05.

### 3.4 Exploratory analysis

Since males accounted for a significantly higher proportion than females in the low-stable group, which may have introduced potential confounding effects, we conducted stratified analyses by gender using group-based trajectory modeling. Compared with males, the proportion of moderate to severe CSVD in females is less. The proportion of various CSVD lesions was also less. After adjusting for the effects of baseline age, alcohol consumption, and education level at baseline, the risk of CSVD was reduced in both the moderate and high stable groups compared with the low-stable group in both males and females. The association between LE8 and lacunar infarcts, CMB, and WMH remained consistent across genders. However, it is noteworthy that compared to the low-stable group, males in the high-stable group exhibited a protective effect against the development of BG-PVS, whereas this association was not observed in females (see Supplementary Tables S3, S4).

We analyzed the effects of health behaviors and health factors trajectories on CSVD separately. The results showed that the influence of health behaviors on CSVD and various lesions of CSVD was not statistically different, while the influence of health factors on CSVD and various lesions of CSVD was greater. However, the combined analysis of health behaviors and factors can reflect the joint effect of these factors on CSVD, which is more helpful to guide people to maintain an ideal healthy life and behavior, so as to reduce the incidence of CSVD (see Supplementary Table S5).

## 4 Discussion

Our findings indicate that there is a negative association between sustained high LE8 scores and the risk of CSVD, with LE8 trajectory demonstrating a stronger impact on CSVD compared to single LE8 scores. Furthermore, the protective effect of high LE8 trajectories appears to be site-dependent, showing more pronounced effects on lacunar infarcts, white matter hyperintensities (WMH), and enlarged perivascular spaces (EPVS).

Since the introduction of the concept of cardiovascular health (CVH), numerous studies have established a link between higher LE8 scores and reduced incidence of cardiovascular diseases. However, there is limited research on the association between LE8 and CSVD, with only one cross-sectional study (17) demonstrating a negative correlation between high LE8 scores and the risk of CSVD. This study found that compared to low LE8 scores (0–60), the odds ratios for developing CSVD in the medium (60–80) and high (80–100) groups were 0.78 (95% CI: 0.63–0.96) and 0.44 (95% CI: 0.33–0.59), respectively. Although our cross-sectional analysis did not find a significant correlation between LE8 scores and CSVD, our study revealed that both the medium and high stable groups had significantly lower risks of elevated CSVD and modified CSVD scores compared to the low stable group. This suggests that maintaining a high level of LE8 can reduce the incidence of CSVD. Cohort studies linking LE8 with stroke or dementia also indirectly support our findings. A study with an average follow-up of 5.65 years (10) showed that participants with ideal LE8 scores (≥80) had a 70% lower risk of ischemic stroke and a 44% lower risk of hemorrhagic stroke compared to those with lower scores (<50). Another cohort study from the UK Biobank (18) confirmed an association between LE8 scores and cognitive function and dementia. Over an average follow-up of 13 years, individuals with optimal LE8 scores (≥80) had a 51% lower risk of mild cognitive impairment (MCI), a 40% lower risk of all-cause dementia, a 54% lower risk of vascular dementia, and a 45% lower risk of non-vascular dementia compared to those with poor LE8 scores (<50). Thus, sustaining high LE8 levels can reduce the risk of CSVD and may even help prevent stroke and dementia.

Our study results indicate that the risk reduction of CSVD associated with high LE8 trajectories also varies by lesion location. The protective effect of high LE8 scores is more pronounced for lacunar infarcts, WMH, and EPVS, while no protective effect was observed for CMB. Although no previous studies have specifically investigated whether cardiovascular health (CVH) protects brain health differently based on lesion location or nature, earlier research has demonstrated that the components of CVH have heterogeneous effects on cerebral small vessel disease. Compared to other factors, hypertension is known to increase the risks of lacunar infarcts, WMH, CMB, and EPVS (19, 20). Secondly, dyslipidemia and obesity may lead to dysregulation of microvascular hemodynamics, thereby increasing blood viscosity and vascular resistance, which subsequently contributes to WMH (21). Some studies support that diabetes is associated with an elevated risk of lacunar infarcts (22). The underlying mechanisms may involve hyperglycemia, insulin resistance, and altered fatty acid metabolism, which induce oxidative stress and activate PKC pathway and the receptor for advanced glycation end products (RAGE). These factors suppress NO production, promote inflammation and thrombogenesis, ultimately causing severe damage to endothelial cells and vascular wall components (23). However, the impact of elevated blood glucose on WMH remains controversial (24). Studies have demonstrated that smoking exacerbates WMH (25), yet its effects on CMB, lacunar infarcts, and EPVS are still debated (26, 27). Proposed mechanisms include the toxic effects of smoking on vascular endothelial cells and its role in activating immune cells and inducing vascular inflammation. Sleep health, as a new component of CVH, significantly influences EPVS. Research indicates that PV, critical pathways for metabolic waste clearance and maintenance of brain interstitial fluid homeostasis, exhibit markedly increased cerebrospinal fluid influx into PVS and interstitial compartments during sleep, facilitating fluid exchange between interstitial and cerebrospinal systems. Sleep disturbances lead to enlarged PVS, reflecting impaired clearance of metabolic waste and brain fluid, thereby aggravating CSVD (28). Additionally, most studies (29, 30) have found no significant correlation between physical exercise and the progression of MRI markers of CSVD. The differing effects of individual LE8 components on lesion location and nature contribute to the variability in LE8’s protective effects on CSVD. However, discussions on CMB remain limited in current research and clinical literature. We should actively explore protective factors that can prevent CMB.

With the rise of unhealthy lifestyles, environmental changes, and an aging population, the incidence of CSVD is rapidly increasing, leading to a corresponding rise in economic burden. Our findings provide valuable evidence for addressing population aging and preventing CSVD.

The limitations of this study include: (1) This study primarily focused on populations in northern China, which has certain limitations. (2) As this study only conducted one cranial MRI examination, we cannot explore the progression of CSVD lesions in relation to LE8 trajectories. (3) We assessed diet through salt intake, tea consumption, and high-fat food consumption, which may partially reflect overall dietary patterns but could overlook the potential impact of other dietary components (such as fresh fruits and red meat). Therefore, caution is warranted in interpreting dietary results. However, our research is ongoing, and we will follow up with the current population and collect detailed information on LE8 to further investigate the impact of LE8 trajectories on the future development of CSVD lesions.

## Data Availability

The raw data supporting the conclusions of this article will be made available by the authors, without undue reservation.
