# Supplementary material for: Sustained high Life’s Essential 8 is associated with lower risk of cerebral small vessel disease: a community-based study
Source: Front Neurol. 2025 Jul 9;16:1563288. doi: 10.3389/fneur.2025.1563288 (PMC12283330; doi:10.3389/fneur.2025.1563288)
Supplement: Supplementary file 1 [file Table_1.DOCX]

**Supplemental Table S1. Definition and scoring approach for quantifying cardiovascular health in the Kailuan study ( Life’s Essential 8 Kailuan Version)***

| Domain | Metric | Method of measurement | Quantification of CVH metric |
| --- | --- | --- | --- |
| Health behaviors | Diet health | Measurement: Self-reported intake of salt, fatty foods, and tea  Examples of salt intake measurement: “What flavor do you prefer.”  Examples of fatty food intake measurement: “How often do you eat fatty foods? ”  Example of salt intake measurement: “How often do you drink tea?” | Metric: The unweighted average of salt, fatty food, and tea scoring.  Salt scoring:  Points Level  100 <6 g/day  50 6-12 g/day   1. >12 g/day   Fatty food scoring:  Points Level  100 <1 time/week  50 1-3 times/week   1. >3 times/week   Tea scoring:  Points Level   1. ≥4 times/week   75 1-3 times/week  50 1-3 times/month  25 < 1 time/month  0 Never |
|  | Physical activity | Measurement: Self-reported times of physical activity per week.  Example tools for measurement: “How many times did you usually spend on physical activity (note: It took at least 20 minutes each time)? ” | Metric: Minutes of physical activity per week.  Scoring:  Points Level  100 ≥ 80  50 20-79  0 <20 |
|  | Nicotine exposure | Measurement: Self-reported use of cigarettes  Example tools for measurement: Do you now smoke cigarettes? (Never smoker, former smoker, some days, every day) | Metric: Smoking status  Scoring:  Points Status  100 Never smoker  50 Former smokers quit ≥ 1 y  25 Current smokers,< 1cigarette/d  0 Current smoker, ≥1cigarette/d |
|  | Sleep health | Measurement: Self-reported average hours of sleep per night  Example tools for measurement: “On average, how many hours of sleep do you get per night?” | Metric: Average hours of sleep per night  Scoring:  Points Level  100 7 - <9 h  90 9 - < 10 h  70 6 - < 7 h  40 5 - <6 or ≥10 h  20 4 - <5 h  0 <4h |
| Health factors | Body mass index | Measurement: Body weight (kg) divided by height squared(m²)  Example tools for measurement: Objective measurement of height and weight | Metric: Body mass index (kg/m^2^)  Scoring:  Points Level  100 <23  75 23.0-24.9  50 25.0-29.9  25 30.0-34.9  0 ≥35.0 |
|  | Blood lipids | Measurement: Plasma total and HDL cholesterol with the calculation of non-HDL cholesterol.  Example tools for measurement: Fasting blood sample.  non-HDL-cholesterol unit conversion:  1mmol/L= 38.67mg/L  1mg/L=0.02586mmol/L | Metric: Non-HDL cholesterol (mmol/L)  Scoring:  Points Level  100 < 3.36  60 3.36-4.13  40 4.14-4.90  20 4.91-5.68  0 ≥ 5.69  If the drug-treated level subtracts 20 points |
|  | Blood glucose | Measurement: Fasting blood glucose(FBG)  Example tools for measurement: Fasting blood glucose sample.  HBA1C to FBG(mg/L) to conversion:  28.7 * A1C - 46.7 = FBG  FBG unit conversion:  1mg = 0.056mmol/L  1mmol/L= 18.02 mg/dL | Metric: FBG (mmol/L)  Scoring:  Points Level  100 No history of diabetes with FBG <5.6  60 No diabetes with FBG 5.6-6.9  40 Diabetes with FBG < 8.6  30 Diabetes with FBG 8.6-10.1  20 Diabetes with FBG 10.2-11.6  10 Diabetes with FBG 11.7-13.2  0 Diabetes with FBG ≥13.3 |
|  | Blood pressure | Measurement: Appropriately measured systolic and diastolic blood pressure  Example tools for measurement: Corrected Mercury sphygmomanometer | Metric: Systolic and diastolic blood pressure (mm Hg)  Scoring:  Points Level  100 <120 / < 80  75 120-129 / < 80  50 130-139 or 80-89  25 140-159 or 90-99  0 ≥160 or ≥100  Subtract 20 points if the treated level |

*Modified from American Heart Association’s New “Life’s Essential 8” Metrics. @Lloyd-Jones, D.M., et al., Life’s Essential 8: Updating and Enhancing the American Heart Association’s Construct of Cardiovascular Health: A Presidential Advisory From the American Heart Association. Circulation, 2022: p. 10.1161/CIR. 0000000000001078.
