# Supplementary material for: Sustained high Life’s Essential 8 is associated with lower risk of cerebral small vessel disease: a community-based study
Source: Front Neurol. 2025 Jul 9;16:1563288. doi: 10.3389/fneur.2025.1563288 (PMC12283330; doi:10.3389/fneur.2025.1563288)
Supplement: Supplementary file 2 [file Table_2.DOCX]

Supplemental Table S2. Magnetic resonance imaging acquisition protocol

| Sequence | Mode | Readout module | Time min:s | TR/TE (ms) | Bandwith | Flip angle (degrees) | Number of slices | Slice thickness(mm) | Spacing between Slices(mm) | FOV (cm2) | Acquisition | Recon | ETL | NEX |
| --- | --- | --- | --- | --- | --- | --- | --- | --- | --- | --- | --- | --- | --- | --- |
| T2WI | 2D | FSE | 1:27 | 5842/103 | 62.5 | 142 | 25 | 5 | 6 | 24×24 | 416×416 | 512×512 | 32 | 1.5 |
| 3D T2WI | 3D | FSE | 4:13 | 2500/84.9 | 62.5 | 90 | 166 | 1 | 1 | 25.6×25.6 | 320×320 | 512×512 | 100 | 1 |
| FLAIR | 3D | FSE | 6:23 | 5000/1147 | 62.5 | 90 | 170 | 1 | 1 | 25.6×25.6 | 256×256 | 512×512 | 160 | 1 |
| BRAVO T1WI | 3D | GRE | 2:34 | 6.7/2.6 | 41.67 | 15 | 170 | 1 | 1 | 25.6×25.6 | 256×256 | 512×512 |  | 1 |
| DWI | 2D | EPI | 0:38 | 5110/77.2 | 250 | 90 | 25 | 5 | 6 | 24×24 | 130×160 | 256×256 |  | 2 |
| SWAN | 3D | GRE | 4:40 | 39.7/23.9 | 41.67 | 15 | 136 | 2 | 1 | 24×24 | 320×320 | 512×512 |  | 0.7 |

Abbreviation: T2WI, T2-weighted imaging; 3D, three-dimensional; FLAIR, fluid-attenuated inversion recovery; BRAVO, brain volume; T1WI, T1-weighted imaging; DWI, diffusion weighted imaging; SWAN, susceptibility-weighted angiography; FSE, fast spin echo; GRE, gradient- recalled echo; EPI, echo-planar imaging; TR, repetition time; TE, Echo time; FOV, field of view; ETL, echo train length; NEX, number of excitation.
