# Supplementary material for: Sustained high Life’s Essential 8 is associated with lower risk of cerebral small vessel disease: a community-based study
Source: Front Neurol. 2025 Jul 9;16:1563288. doi: 10.3389/fneur.2025.1563288 (PMC12283330; doi:10.3389/fneur.2025.1563288)
Supplement: Supplementary file 3 [file Table_3.DOCX]

| Supplemental Table S3 Distribution of CSVD and Various CSVD Lesions | | | | | | |
| --- | --- | --- | --- | --- | --- | --- |
|  | Variables | Total | Low-stable group | Medium-stable group | High-stable group | *P* |
| Male |  |  |  |  |  |  |
|  | N | 575 | 128 | 314 | 133 |  |
|  | CSVD score |  |  |  |  | 0.011 |
|  | 0 | 107 (18.6) | 19 (14.8) | 52 (16.6) | 36 (27.1) |  |
|  | 1 | 186 (32.3) | 38 (29.7) | 104 (33.1) | 44 (33.1) |  |
|  | 2 | 137 (23.8) | 28 (21.9) | 78 (24.8) | 31 (23.3) |  |
|  | 3 | 83 (14.4) | 24 (18.8) | 41 (13.1) | 18 (13.5) |  |
|  | 4 | 62 (10.8) | 19 (14.8) | 39 (12.4) | 4 (3.0) |  |
|  | Lacunar infarcts | 136 (23.7) | 43 (33.6) | 77 (24.5) | 16 (12.0) | <0.001 |
|  | CMB | 205 (35.7) | 53 (41.4) | 107 (34.1) | 45 (33.8) | 0.304 |
|  | WMH | 178 (31.0) | 41 (32.0) | 108 (34.4) | 29 (21.8) | 0.030 |
|  | BG-PVS | 438 (76.2) | 105 (82.0) | 247 (78.7) | 86 (64.7) | 0.001 |
| Female |  |  |  |  |  |  |
|  | N | 587 | 145 | 271 | 171 |  |
|  | CSVD score |  |  |  |  | <0.001 |
|  | 0 | 192 (32.7) | 24 (16.6) | 86 (31.7) | 82 (48.0) |  |
|  | 1 | 238 (40.5) | 48 (33.1) | 116 (42.8) | 74 (43.3) |  |
|  | 2 | 100 (17.0) | 39 (26.9) | 48 (17.7) | 13 (7.6) |  |
|  | 3 | 37 (6.3) | 19 (13.1) | 16 (5.9) | 2 (1.2) |  |
|  | 4 | 20 (3.4) | 15 (10.3) | 5 (1.8) |  |  |
|  | Lacunar infarcts | 48 (8.2) | 31 (21.4) | 17 (6.3) |  | <0.001 |
|  | CMB | 127 (21.6) | 47 (32.4) | 51 (18.8) | 29 (17.0) | 0.001 |
|  | WMH | 122 (20.8) | 55 (37.9) | 50 (18.5) | 17 (9.9) | <0.001 |
|  | BG-PVS | 332 (56.6) | 110 (75.9) | 162 (59.8) | 60 (35.1) | <0.001 |
| Lacunar infarcts was classified into grade 0-1; CMB: cerebral microbleeds, classified into grade 0-1; WMH:white matter hyperintensities, classified into grade 0-1; BG-EPVS：enlarged perivascular space in basal ganglia,classified into grade 0-1. | | | | | | |

| Supplemental Table S4 Impact of LE8 Trajectory Groups on CSVD and Various CSVD Lesions | | | | | |
| --- | --- | --- | --- | --- | --- |
|  | Groups | Male |  | Female |  |
|  |  | OR (95%CI) | *P* | OR (95%CI) | *P* |
| CSVD score |  |  |  |  |  |
|  | Low-stable | 1.00 |  | 1.00 |  |
|  | Medium-stable | 0.66(0.45-0.96) | 0.031 | 0.66(0.44-0.99) | 0.044 |
|  | High-stable | 0.43(0.27-0.69) | <0.001 | 0.61(0.38-1.01) | 0.052 |
| Lacunar infarcts |  |  |  |  |  |
|  | Low-stable | 1.00 |  | 1.00 |  |
|  | Medium-stable | 0.46(0.27-0.78) | 0.004 | 0.38(0.19-0.74) | 0.005 |
|  | High-stable | 0.2(0.09-0.41) | <0.001 | <0.001(<0.001->999.999) | 0.94 |
| CMB |  |  |  |  |  |
|  | Low-stable | 1.00 |  | 1.00 |  |
|  | Medium-stable | 0.67(0.43-1.05) | 0.078 | 0.67(0.41-1.1) | 0.116 |
|  | High-stable | 0.74(0.43-1.27) | 0.275 | 0.84(0.45-1.58) | 0.585 |
| WMH |  |  |  |  |  |
|  | Low-stable | 1.00 |  | 1.00 |  |
|  | Medium-stable | 1.02(0.62-1.66) | 0.95 | 0.62(0.37-1.02) | 0.058 |
|  | High-stable | 0.6(0.32-1.14) | 0.119 | 0.61(0.3-1.25) | 0.175 |
| BG-PVS |  |  |  |  |  |
|  | Low-stable | 1.00 |  | 1.00 |  |
|  | Medium-stable | 0.77(0.43-1.39) | 0.383 | 0.98(0.57-1.69) | 0.948 |
|  | High-stable | 0.38(0.2-0.75) | 0.005 | 0.69(0.38-1.26) | 0.23 |
| Lacunar infarcts was classified into grade 0-1; CMB: cerebral microbleeds, classified into grade 0-1; WMH:white matter hyperintensities, classified into grade 0-1; BG-EPVS：enlarged perivascular space in basal ganglia,classified into grade 0-1. | | | | | |
