# Supplementary material for: Sustained high Life’s Essential 8 is associated with lower risk of cerebral small vessel disease: a community-based study
Source: Front Neurol. 2025 Jul 9;16:1563288. doi: 10.3389/fneur.2025.1563288 (PMC12283330; doi:10.3389/fneur.2025.1563288)
Supplement: Supplementary file 4 [file Table_4.DOCX]

| Supplemental Table S5 Impact of Health behaviors and Health factors Trajectory Groups on CSVD and Various CSVD Lesions | | | | | |
| --- | --- | --- | --- | --- | --- |
|  | Groups | Health behaviors | | Health factors | |
|  |  | OR (95%CI) | *P* | OR (95%CI) | *P* |
| CSVD score |  |  |  |  |  |
|  | Low-stable | 1.00 |  | 1.00 |  |
|  | Medium-stable | 0.73(0.52-1.03) | 0.07 | 0.49(0.37-0.64) | <0.001 |
|  | High-stable | 0.87(0.62-1.23) | 0.43 | 0.41(0.3-0.57) | <0.001 |
| Lacunar infarcts |  |  |  |  |  |
|  | Low-stable | 1.00 |  | 1.00 |  |
|  | Medium-stable | 0.67(0.41-1.08) | 0.101 | 0.41(0.28-0.59) | <0.001 |
|  | High-stable | 0.71(0.42-1.17) | 0.179 | 0.2(0.1-0.39) | <0.001 |
| CMB |  |  |  |  |  |
|  | Low-stable | 1.00 |  | 1.00 |  |
|  | Medium-stable | 0.98(0.66-1.47) | 0.93 | 0.68(0.5-0.94) | 0.018 |
|  | High-stable | 0.99(0.65-1.49) | 0.942 | 0.76(0.51-1.13) | 0.173 |
| WMH |  |  |  |  |  |
|  | Low-stable | 1.00 |  | 1.00 |  |
|  | Medium-stable | 0.73(0.47-1.14) | 0.164 | 0.58(0.42-0.82) | 0.002 |
|  | High-stable | 0.96(0.62-1.51) | 0.869 | 0.65(0.42-1.01) | 0.053 |
| BG-PVS |  |  |  |  |  |
|  | Low-stable | 1.00 |  | 1.00 |  |
|  | Medium-stable | 0.77(0.47-1.26) | 0.299 | 0.55(0.37-0.83) | 0.005 |
|  | High-stable | 0.86(0.53-1.4) | 0.537 | 0.37(0.23-0.58) | <0.001 |
| Lacunar infarcts was classified into grade 0-1; CMB: cerebral microbleeds, classified into grade 0-1; WMH:white matter hyperintensities, classified into grade 0-1; BG-EPVS：enlarged perivascular space in basal ganglia,classified into grade 0-1. | | | | | |
